# GTPases and the origin of the ribosome

**DOI:** 10.1186/1745-6150-5-36

**Published:** 2010-05-20

**Authors:** Hyman Hartman, Temple F Smith

**Affiliations:** 1 Center for Biomedical Engineering, Massachusetts Institute of Technology, Cambridge, MA 02139, USA; 2 BioMolecular Engineering Research Center, Boston University, 36 Cummington Street, Boston, MA 02215, USA

## Abstract

**Background:**

This paper is an attempt to trace the evolution of the ribosome through the evolution of the universal P-loop GTPases that are involved with the ribosome in translation and with the attachment of the ribosome to the membrane. The GTPases involved in translation in Bacteria/Archaea are the elongation factors EFTu/EF1, the initiation factors IF2/aeIF5b + aeIF2, and the elongation factors EFG/EF2. All of these GTPases also contain the OB fold also found in the non GTPase IF1 involved in initiation. The GTPase involved in the signal recognition particle in most Bacteria and Archaea is SRP54.

**Results:**

1) The Elongation Factors of the Archaea based on structural considerations of the domains have the following evolutionary path: EF1→ aeIF2 → EF2. The evolution of the aeIF5b was a later event; 2) the Elongation Factors of the Bacteria based on the topological considerations of the GTPase domain have a similar evolutionary path: EFTu→ IF→2→EFG. These evolutionary sequences reflect the evolution of the LSU followed by the SSU to form the ribosome; 3) the OB-fold IF1 is a mimic of an ancient tRNA minihelix.

**Conclusion:**

The evolution of translational GTPases of both the Archaea and Bacteria point to the evolution of the ribosome. The elongation factors, EFTu/EF1, began as a Ras-like GTPase bringing the activated minihelix tRNA to the Large Subunit Unit. The initiation factors and elongation factor would then have evolved from the EFTu/EF1 as the small subunit was added to the evolving ribosome. The SRP has an SRP54 GTPase and a specific RNA fold in its RNA component similar to the PTC. We consider the SRP to be a remnant of an ancient form of an LSU bound to a membrane.

**Reviewers:**

This article was reviewed by George Fox, Leonid Mirny and Chris Sander.

## Background

This study is on the origin and evolution of the ribosome Large Subunit (LSU) and Small Subunit (SSU) as reflected in the structure of the universal ribosomal translational GTPases. This continues an earlier study [1] on the origin of the ribosome as reflected in the structure of the ribosomal proteins. That study led to the conclusion that the LSU began as a Peptide Transfer Center (PTC)-like RNA fold on a peptide membrane. The subsequent evolution of the LSU was only later joined by the addition of an evolving SSU.

Recent papers by Bokov and Steinberg [2], Hury *et al*. [3], and Fox and Naik [4] consider the evolution of the LSU of the ribosome to start from a PTC-like RNA. It is becoming clear that the evolution of the ribosome begins with the evolution of the LSU. This should be reflected in the evolution of the GTPases associated with the ribosome, especially those common to both the Archaea and the Bacteria.

The translational GTPases are the Bacteria elongation factors, EFTu, EFG, and the initiation factor, IF2, and their archaeal homologs, the EF1, EF2, aeIF5b and aeIF2. There are also two additional GTPases associated with transmission of the translated peptide chain from the ribosome to the membrane, the Ffh/SRP54 as part of the signal recognition particle (SRP) and the GTPase, FtsY/SR-alpha, which is attached to the cell membrane.

The initiation factors are involved with bringing the f-Met-charged tRNA to the SSU ribosomal subunit and the joining of the SSU and LSU ribosomal subunits. The elongation factors, EFTu/EF1, bring the next charged tRNA to the ribosome and interact primarily with the large subunit, while EF2/EFG initiate the elongation cycle involving the PTC peptide bond formation along with the allosteric motion of the whole ribosome.

**Figure 1:**
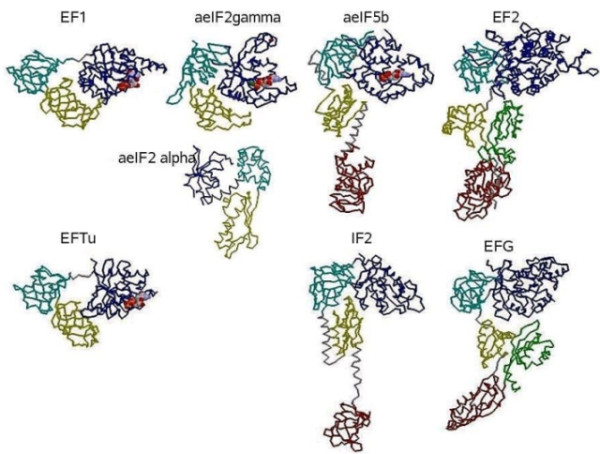
**The peptide backbones of the archaeal translational proteins as represented in the protein structure database, PDB, by the following ID codes and displayed in RasMol**. These are: 1SKQ (EF1), 1KK3 (IF2 gamma), 2AHO (IF-2 alpha), 1G7S (aIF5b) and 1NOU (EF2) and the bacterial counterparts, 1EFC (EFTu), 1ZO1 (IF2) and 1PN6 (EFG).

EF1, aeIF5b, aeIF2 and EF2 from Archaea and their bacterial counterparts, EFTu, IF2 and EFG, are multidomain proteins. They all contain two homologous N-terminal domains: a GTPase or G-domain, followed by an OB-domain [5]. The peptide backbones of these proteins are displayed in Figure 1. For ease of comparison, these proteins' domains are colored consistently in the figure from their N-terminal to C-terminal. The N-terminal G-domain is dark blue and thesubsequent domains are: cyan for the second domain, yellow for the third, red for the fourth and green for the fifth. The same coloring is also used for the aeIF2 alpha subunit of the archaeal aeIF2 complex. Here aeIF2 complex refers to the functional complex formed by aeIF2 gamma and aeIF2 alpha, which is a second initiation component along with aeIF5b in Archaea, but is not found in Bacteria.

In addition to the common first two domains, archaeal EF1and bacterial EFTu have a third C-terminal OB-domain (yellow) in common. IF2 and aeIF5b have two additional domains linked by long helices. The domain three of the bacterial IF2 is a simple alpha beta sandwich, while domain four is another OB beta barrel, similar to IF1 and to the second domain of EFTu/EF1.

These translational proteins' G-domains are both structurally and functionally related to a larger family of GTPase G proteins [6]. All contain a common core with a Walker A motif or P-loop associated with the nucleotide and phosphate binding. This characteristic motif is located between the fold's initial beta strand and the adjoining alpha helix. Following this first helix is the so-called switch region [7], which is imbedded in an overall central beta alpha/beta fold. An accepted prototypic G-domain is found in Ras P21 (pdb ID, 5P21[8]). The secondary structure topology of the EFTu G-domain is identical to such a prototypic G protein. The analysis in this study of the translational GTPases supports the co-evolution of these proteins with that of the ribosome. Beginning from a simple peptidyl transferase center (PTC) interacting with a Ras-like G-domain, a small OB-domain and mini aminoacylated RNA helices, the evolution would have progressed with the addition to the early EFTu, of IF2 and EFG as the small ribosomal subunit.

## Results

### GTPase Domains

**Figure 2:**
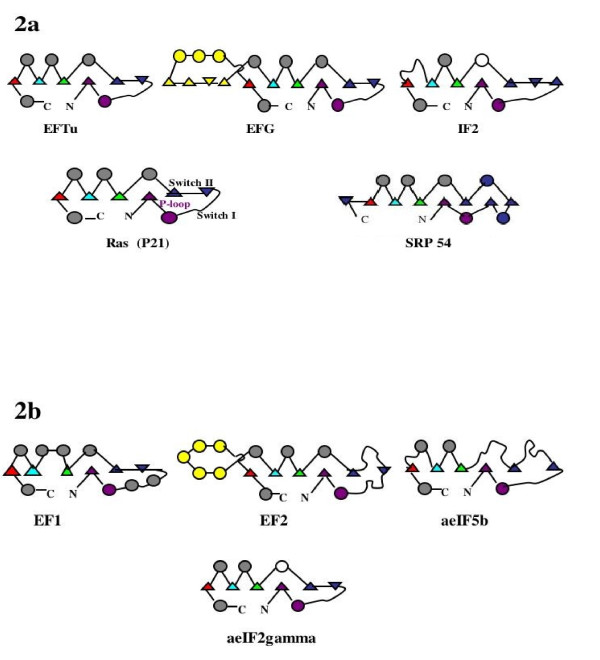
**The translational protein G-domain secondary structure topologies**. 2a displays the G-domain topologies for the bacterial proteins plus Ras and SRP54. 2b displays those for the archaeal translational proteins. These are based on the same PDB data represented in Figure 1, with the addition of 5P21 for Ras and 1J8M for SRP54. The topology of SR-alpha, and the bacterial Ffh and FtsY are not shown as they are nearly identical to that of SRP54. The triangles represent beta strands; the circles represent alpha helices. The region of the P-loop motif adjoining strand and helix are colored purple for reference. The highly variable switch region beta strands are colored blue. The yellow represents the large insertions in EF2/EFG relative to Ras. The white circles represent the significant length reduced helices in the bacterial IF2 and the archaeal aeIF2 gamma.

Comparison of the other translational protein G-domains with the structural topology ofRas or EFTu allows the identification of their non-core differences. For example, IF2 and EFG contain a number of non-core modifications (Fig. 2). EFG has a significant structural insertion near the C-terminal of the G-domain. On the other hand, IF2 has two structural reductions relative to Rasand EFTu: a reduced alpha helix two and the replacement of helix four by a disordered loop.

**Figure 3:**
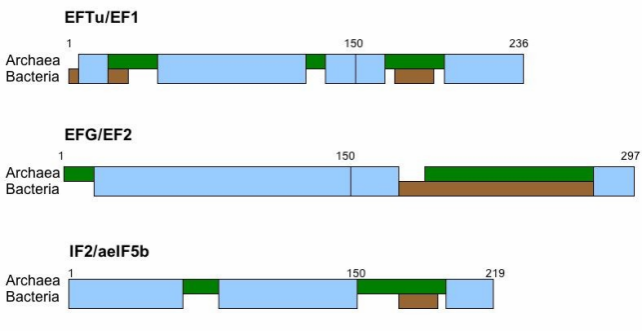
**Translational G-protein taxonomic division sequence block alignments**. Structure based aligned sequences: Numbering is over total aligned set of Archaea *(S. solfataricus, M. sedula, H. butylicus, M. jannaschii, and T. thermophilus) *and three bacteria *(G. stearothermophilus, B. halodurans, E. coli)*. (A) Displays the alignments for EFTu/EF1, (B) those of IF2/aeIF5b and (C) those of EFG/EF2. Light blue indicates sequence segments or universal blocks that can be aligned across both taxonomic divisions having similar secondary structures and with statistical significance; Green indicates sequence blocks alignable among and unique to Archaea, while brown indicates blocks alignable among and unique to Bacteria. These definitions are consistent with those used in Smith 2008 [1].

The structural and functional similarity among these universal G-domain translation factors reveals their common GTPase ancestry. For example, at the sequence level the G-domains of the homologs EF1and EFTu can be aligned, revealing regions of obvious sequence conservation in spite of some structural differences, as seen in Figure 2. However, those same alignments display a set of phylodomain-specific sequence segments or blocks, similar to those seen in comparisons between bacterial and archaeal ribosomal proteins [9], see Figure 3. This same mixture of alignable and nonalignable segments is seen by Gribaldo and Cammarano [10]in their phylogenetic analysis of EFG with EF2.

The universal ribosomal protein sequences display phylodomain block structure [9], that is, their amino acid sequences display three distinct segments or blocks of amino acids: one, universal sequence blocks common to both Bacteria and Archaea; two, blocks common and unique to all Bacteria; and, third those common and unique to all Archaea. Such sequence block structure has implied the existence of three last common ancestors: a common ancestor of both Bacteria and Archaea based on the universal blocks, and two subsequent last common ancestors, one for the Bacteria based on the bacterial-specific blocks and a last common ancestor for the Archaea based on the archaeal-specific blocks.

TheEFTu/EF1 G-domain alignments show a 24-amino acid archaeal unique block juxtaposed to a short ten-amino acid bacterial unique block. The length difference can be identified with the additional helices seen in Figure 2 in the so-called switch one region. There are two other archaeal-specific blocks, the second one slightly longer than its juxtaposed bacterial distinct sequence block. An examination of the comparative sequence alignments of the translational proteins EFG with the archaeal EF2shows similar taxonomic division-specific blocks within their G-domains. Here the large C-terminal G-domain insertions are of different lengths and clear distinct sequences forming their juxtaposed phylodomain-specific block.

**Figure 4:**
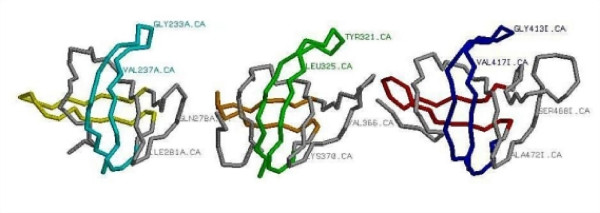
**Structure similarities between the bacterial EFTu, EFG and IF2, OB second domains**. Here are displayed three nearly identical substructures within three OB-domains: one in yellow, orange and red and the second in cyan, green and blue. The lack of any significant sequence similarity among the clear homologues is indicated by the very different amino acids at the marked equivalent labeled positions.

In the case of IF2 there are two blocks found in the archaeal G-domain as inserts. The first of these appears as an insertion in the highly variable switch II region of the second and third beta strands. The second archaeal block is juxtaposed to a shorter bacterial region containing a near complete deletion of the bacterial fourth alpha helix (Figure 3 and 4).

A comparison across all of these GTPases reveals high sequence and structural variability in the switch region. In particular there is a decrease in secondary structural organization as one moves from EF1 to aeIF5b in Archaea, and to a lesser extent in Bacteria from EFTu and EFG to IF2.

The last helix of all of the G-domains is also highly variable in sequence and slightly different in length between and within the bacterial and archaeal in all three of the above translational factors. Given the somewhat unconstrained nature of this region as a domain-connecting substructure, they are not considered a phylodomain **-**specific block of the same character as those above. This variation is unlikely to have a modulating affect on GTPase activity.

### The Signal Recognition Particle (SRP) GTPases

Approximately one-third of the proteins of a cell are found in membranes. The ribosome must get to the membrane as these membrane proteins are synthesized on the membrane. This process is mediated by the SRP, which is a complex of a small RNA and a small set of proteins. The SRP attaches to a signalhydrophobic leader peptide emanating from the large ribosomal subunit. SRP54 is the universal GTPase on the SRP that binds the leader sequence and subsequently forms a symmetric heterodimer with the membrane-bound universal SR GTPase. This heterodimer, which is attached to the ribosome, then hydrolyses the bound GTP resulting in the attachment of the ribosome to the membrane.

The GTPase associated with the membrane and that interacts with the SRP is the FtsY (SR-alpha in Archaea) protein. This protein has two domains, G domain (GTPase) and an N-terminal domain, a four-helical bundle. The G-domain belongs to the SIMBI class of the P-loop GTPases [6]. The archaeal SR-alpha has been shown to be very similar in structure to the bacterial FtsY[11]. In addition the FtsY is closely related to the Ffh protein of bacterial SRP and to the Ffh archaeal homolog, SRP54 [12,13]. Gribaldo and Cammarano rooted the cellular tree on the branch leading to Ffh and SRP54 [10]. The Ffh and SRP54 have a third three-C-terminal domain, the M domain. This third domain of Ffh and SRP54 is involved in binding to the peptide sequence protruding from the LSU [14]. The high sequence and structural conservation of this SRP GTPase coupled with the conservation of their RNA binding sites supports an extreme age for these proteins and the helices of the SRP's RNA [15,16].

### OB-Domain

All seven of the translational GTPases displayed in Figure 1 have a small beta barrel OB-domain as their second domain. In 1993 the OB fold was first characterized by Murzin analyzing the structure of four proteins (two bacterial toxins, aspartyl tRNA synthetase and a nuclease). It is a "five-stranded beta sheet coiled to form a closed beta-barrel. This barrel is capped by an alpha-helix located between the third and fourth strands", but "no similarities can be observed in the corresponding alignment of the four sequences" [17].

More recently the OB fold has been reviewed by Arcus describing the structure and function of OB fold domains [5]. Among the characteristic properties of the OB fold was its binding to single-stranded nucleic acids. The ribosomal proteins, S1, S12, S17, L2 and IF1, are among those ribosomal proteins with OB-domains that have been shown to bind single-stranded RNA [18]. The RNA interaction of the OB-domain is of special interest among the proteins considered in this paper since a number of them have an OB-domain that interacts with the RNA. IF1 is composed only of an OB fold and interacts with the small ribosomal subunit, especially with the bases A 1492 and A 1493. EFTu has three domains, the second being an OB fold that interacts with the CCA of the tRNA [19]. IF2 is a multiple domain protein having an OB fold as the fourth domain, which also interacts with the CCA of the tRNA [20]. We believe that this OB-RNA interaction is a remnant of a very ancient interaction between early peptides and RNAs.

There is a weak sequence similarity between the OB folds of EFTu and EF1. However there are no significant sequence similarities among the other GTPase OB second domains. There is a 14-15 amino acid insert at the very start of the OB second domain in EFG that has no equivalent in the archaeal EF2. A pure structural comparison between the bacterial and archaeal homologs of these OB-domains displays considerable similarity. For example the structures of the three bacterial translational GTPase OB second domains show remarkable similarities, see Figure 4.

Interestingly the structure similarity of these proteins' second domains carries over to the fourth domain of the IF2's and the entire structure of the IF1's. In the two latter cases the OB-domain likely interacts directly with either the tRNA or the active site of the small ribosomal subunit mRNA [21]. In the IF2 case it would be with the CCA of the initiating tRNA. In theIF1 case it binds in the ribosomal A site and binds the bases, A1492 and A1493, from Helix 44 [22].

As noted above there are very similar OB-domains found in a wide range of protein families, including the translation-related ribosomal protein, S1, containing six such domains. As with EFTu/EF1 and IF2, the second OB-domain of S1 has been shown to directly bind single-stranded RNA as it delivers transfer-messenger RNA (tmRNA) to stalled ribosomes [23]. This, combined with the ideas of the very early role for RNA in the evolution of the translational apparatus, suggests that such OB-domains may represent one of the oldest such binding domains.

There are additional small beta barrel OB-like domains in the EFTu/EF1 and EFG/EF2 homologs as their third domain. These have less bacterial-to-archaeal sequence similarity than the second domains, but with evidence of at least two significant taxonomic domain blocks. A curious fact is that these third domains of EFTu/EF1 and EFG/EF2 are wound in the reverse direction to their common second domains. The reversed OB fold of this third domain interacts with the doubled stranded RNA of the CCA arm of the tRNA [24].

### Additional Domains of IF2

Connected to the bacterialIF2'sOB second domain via a six-turn helix is a small alpha beta domain that appears to make contact with both the G-domain and the OB second domain. Then there a fourth domain with an OB-fold, connected by a long helix. The latter has a set of loops at the far extremity of the protein that, like other OB **-**domains [18], may make RNA connects such as the CCA of the incoming tRNA. The archaeal IF2is different in a number of ways. Its OB second domain is connected to a very different third domain of five beta strands and a single helix. Then the similarity with the bacterialIF2 resumes with a long connecting six-turn helix with its fourth domain, which is again an OB-domain, slightly distorted with two added short alpha helices.

### Additional Domains of EFG/EF2

Moving beyond the common G-domains and OB-domain of the EFG/EF2 proteins, there are three more domains. In the bacterial case these form a structure spatially similar to a tRNA [19]. The structure of domain four of EFG is composed of a small alpha beta domain of four beta strands and two helices followed by an elongated larger four-stranded alpha beta domain extending away from the rest of the structure. This is followed by the protein's fifth domain, a small somewhat disordered alpha beta domain that packs with the protein's third domain and its G-domain. While there is no high resolution determined archaeal EF2 structure, the sequence alignments with both the Bacteria and yeast EF2s support a rather similar structure with minor sequence insertions.

## Discussion

In an earlier paper [1], a model was proposed for an early form of a ribosome that began as a self-folding RNA attached to a membrane formed from short peptides. This self-folding RNA was the precursor to the modern peptidyl transferase center (PTC) and was composed of (three) RNA helices similar to those found at the extant PTC. This modeled RNA membrane-bound structure catalyzed the peptidyl transfer reaction from the ancient charged tRNAs, which were mini RNA helices. This would mean that the ribosome began as a PTC RNA membrane-bound structure and evolved into the LSU, with the evolving SSU added later.

The current analysis of the translational GTPases, including the SRP and SR GTPases, provides additional support for such a model. It was noted by Montoya *et al*., "The sequence similarity between Ffh and FtsY and the high number of known sequences allow us to perform phylogenetic studies on the SRP-GTPase family. These suggest that the SRP-GTPases might have evolved earlier than the small GTPases" [13]. One consequence of this finding is that the GTPase associated with the 7SL RNA of the SRP may be considered an earlier evolutionary form of the GTPases that evolved into the Ras-like GTPases found among the extant translational proteins. What may be of further interest is that the two looped helices of the extant 7SL SRP RNA are similar in structure to the two side-by-side looped RNA helices found in the PTC of the LSU and essential to the earlier proposed PTC model [1]. They were included in our proposed model for the early PTC. A remnant of the ancient proto ribosome, we believe, may be found in the SRP.

### The translational GTPases

The common ancestor of the translational GTPases began with a Ras-like GTPase to which an OB fold was joined. This ancient elongation factor was the precursor to EFTu/EF1. The ancestor of the EF2/EFG and IF2 would have come later, evolving from the first two EF1/EFTu domains as the SSU mRNA complex began to interact with the LSU peptidyl transfer center. This would have been coordinated with the evolution of the full tRNA structure. The evolution of the molecular mimicry by EFG of the EFTu tRNA complex, as noted earlier by Fox and Naik [4], reflects this sequence of events in the archaeal IF2 gamma (Figure 2).

The IF1 as an OB fold is a small protein (70 to 100 amino acids) and binds to a single stranded RNA. This fold is found in a number of ribosomal proteins. The extant IF1 as a single OB fold appears to block the A-site, forcing initiation to begin in the P-site. Thus this OB fold appears capable of mimicking the anticodon stem loop of an ancient tRNA. An original form of the IF1 OB fold could have been used to modulate peptide formation at the proto-PTC site of the evolving large ribosomal subunit. A later fusion with a Ras-like GTPase then led to the formation of EFTu and the controlled delivery of the activated mini tRNAs to the PTC of the evolving LSU.

One further consequence of this evolution of the ancestor of EFTu/EF1 is that it must have been connected with the evolution of the transfer RNA itself. In a previous paper [25] on the evolution of tRNA, the candidate for the ancient tRNA was proposed to be an oligonucleotide, 21 nucleotides with a seven-nucleotide loop attached to short double helical ending in a "discriminator" nucleotide and CCA. It was a model of the anti-codon arm to which a CCA was added, or alternatively that of an acceptor arm [25]. Such a model for the ancient tRNA became an experimental subject when Schimmel and his students [26] began a series of studies on the aminoacylation of RNA minihelices by aminoacyl tRNA synthetases. The RNA minihelices were a model of the presumed acceptor stem of an ancient tRNA. They found that the aminoacyl tRNA synthetases, especially that of the alanine and glycine tRNA synthetases, would activate the amino acid and then add it to the CCA of these minihelices.

The OB fold may reflect this evolution of the ancient tRNA as the OB fold can be viewed as a molecular mimic of the anticodon stem of the ancient tRNA, e.g., IF1. The OB fold may also have interacted with the CCA end of the early minihelical tRNAs, as in the extant EFTu. The third domain of EFTu, the reverse OB fold, would have been added to interact with the helical portion of the ancient tRNAs for stabilization purposes. These interactions are seen in the extant EFTu tRNA complex [27].

The Archaea, as has been noted, have an initiation factor IF2 complex that has two separate protein subunits: the gamma composed of a G-Domain and two OB folds, and a second, alpha, subunit composed of a three-domain subunit similar to those of the three C-terminal domains of the bacterial EFG (Fig. 1). The Archaea have a second initiation factor, aIF5b, that is a homolog to the bacterial initiation factor, IF-2. Since the bacterial initiation system has only one initiation factor, it is thus a simplification of the archaeal system of initiation. Furthermore since there are fewer ribosomal proteins associated with the bacterial ribosome (57 ribosomal proteins) than the archaeal ribosome (68 ribosomal proteins) and the simpler system of GTPases associated with the bacterial ribosome, it is reasonable to conclude that the bacterial ribosome is a streamlined archaeal ribosome.

The universal sequence blocks found in the translational G-domains imply that modern Bacteria and Archaea (as defined by their translational machinery) clearly had a common ancestor. Yet the phylodomain-specific blocks imply that the two groups each derive from single, phylodomain-specific types that came into existence some point long after that common ancestor with a fully functional translational system. Why only two basic types? One simple explanation proposed is a major evolutionary bottleneck, drastically limiting the progenitors of modern cell domains [9].

Another hypothesis to consider in the evolution of the translational apparatus is that the earliest translational protein precursors were conglomerates of peptides that only later were amalgamated into a single sequence of amino acids. For example, the universal sequence blocks of the G-domains may be remnants of an earlier peptide form of these proteins. The OB-domain is an obvious example as it can be assembled from multiple copies of such a short peptide that could form a stable beta hairpin. The idea of short peptides interacting with RNA at a very early time is consistent with the proposal that the earliest form of the proto-ribosomal peptidyl transfer center was composed of a few RNA helices stabilized on a peptide membrane [1].

## Conclusions

The evolution of the extant ribosome-associated GTPases would have begun with a fusion of an OB fold to a Ras-like GTPase whose function was to transport aminoacyl ancestral tRNAs to the membrane-associated self-folding PTC RNA. The initiation and elongation factors evolved from this EFTu/EF1 by adding modules that allowed these GTPases to interact with the SSU. This is consistent with our original hypothesis that the PTC domain of the ribosomal LSU evolved first.

## Methods

The various sequence databases (NCBI, Swiss-Prot, ProDom) and structural databases (RCSB and PDB) were searched for a wide range of bacterial and archaeal representatives of the translational GTPase and related proteins. These were examined for their range of variation, and representatives selected. Then using standard sequence comparison methods [28] and structural alignments [29], initial sequence and structural alignments were generated. These in turn were carefully refined by-hand to ensure that homologous domains and functional sites were properly aligned and superimposed. This allowed the identification of taxonomic-specific blocks to be identified, as in Vishwanath *et al*. [9]. In addition, the secondary structural elements displayed in Figure 2 were extracted from these compared structures. These were then mapped using the notation from Westhead *et al*. [30]. The identification of the different protein domains was largely based on the literature cited from earlier studies of these protein's structures and from the structural alignments. These allowed the clear identification of shared substructures that, in turn, implied shared domains and shared history. All of this used the various protein structure display tools, RasMol [31] and PyMol [32], both for the visual analysis and the generation of Figures 1 and 3.

## Competing interests

The authors declare that they have no competing interests.

## Authors' contributions

TFS designed the study, performed the research and helped to draft the manuscript. HH designed the study, performed the research and helped to draft the manuscript. All authors read and approved the final manuscript.

## Reviewers' Comments

### George Fox

(1) This manuscript is a significant contribution to the study of ribosome evolutionary history by providing strong evidence for the relative age of the main P-loop GTPases using comparisons of structures and sequences of the various molecules under consideration. It is not clear why EF-P whose structure closely resembles eIF5A (Blaha et al., Science 325:966-970, 2009) is not considered or even mentioned. It is especially interesting to learn that the GTPase Domain produces recognizable sequence blocks, which suggests largely divergent evolution. With regard to these blocks, it would be appropriate to add the sequence alignments underlying Figure 3 as supplementary material. In contrast it is found that the OB fold that comprises Domain 2 does not produce recognizable sequence blocks. This implies that Domain 2 of the various proteins under consideration had separate evolutionary origins, e.g. likely recruited from different places, and it will in the future (not required here) be interesting if these origins can be identified. In summary this important paper should be published largely as is, pending possible revisions as detailed below.

Authors' response: We have focused this paper only on those ribosomal GTPases that are universal both to Bacteria and Archaea. EF-P and eIF5A do have an OB fold similar to IF1 and the GTPase second domains, thus we have added them to the list in our discussion on the OB folds.

Minor points (suggested but not required revisions):

(1) Suggest rotating aeIF2 alpha image in Figure 1 by 180 degrees so as to more closely resemble views given for other structures.

Authors' response: This would have required a complete redo. We decided it was only a suggestion.

(2) In Figure 2 the caption should include explanation of circle and triangle symbols for those readers who may not be familiar with this notation.

Authors' response: Done

(3) In the third paragraph of Background section initiation factor text is confusing- suggest ".... EF-Tu, EFG, IF2, and their archaeal homologs EF-1, EF2, aeIF5b and aeIF2."

Authors' response: Rewritten as suggested.

(4) In the fourth paragraph of Background section the phrase "while EF2/EFG initiate the elongation cycle via the PTC peptide bond formation." Is unclear and should be rephrased.

Authors' response: We rephrased to clarify.

(5) In the sixth paragraph of Background section suggest revised wording "".... similar to the second domain of EFTu/EF1 and IF1." In addition, the phrase "not shown" removed because it is not clear what is not shown.

Authors' response: Done.

(6) In the fifth paragraph of the Results section "with no equivalent bacterial segments" is not true of the second block where the bacterial segment although much smaller than the archaeal segment is nevertheless of similar size as that found in Bacterial EFTU/EF1 and therefore is probably not appropriately referred to as "apparently almost complete deletion". Suggest saying that first has no equivalent and then discussing second separately.

Authors' response: Rewritten as suggested.

(7) In the first paragraph of the Discussion section the following language might be better--""This RNA IS ENVISIONED AS membrane-bound structure catalyzed the peptide transfer reaction from the ancient charged tRNAs, which were mini RNA helices. This HYPOTHESIS IMPLIES that"

Authors' response: Done.

(8) In the first paragraph of the subsection entitled "The translational GTPases" it should be noted that Fox & Naik have previously pointed out that molecular mimicry suggests that the EF-Tu-tRNA complex may have preceded EFG.

Authors' response: Reference added.

(9) In the second paragraph of the subsection entitled "The translational GTPases" the phrase "The IF1 as an OB fold" is unclear- perhaps what is meant is something like "The OB domain found in IF-1 is effectively a small protein etc "

Authors' response: As noted below in response to the second reviewer, the entire OB section has been rewritten, and we have also clarified the single OB domain nature of IF1.

### Leonid Mirny

The manuscript presents a phylogenetic and structural analysis of bacterial and archaeal translation-associated GTPases. The authors point at several regions and domains of bacterial and archaeal GTPases that are structurally similar, while having little similarity in sequence. Using this analysis authors conclude that the earliest event in the evolution of ribosome-associated proteins was the fusion of OB-fold domain with Ras-like GTPase domain. They also suggest that OB-fold mimics ancient tRNA minihelix. This is a very interesting study with results shining light on one of the oldest and most fundamental events in the evolution of life on earth.

Results section of the manuscript presents extensive structural analysis that is very hard to follow. My recommendation is to develop a table that summarizes authors' observation on domain-by-domain bases. Numerous three-letter acronyms should also be summarized in a single place in the manuscript.

My understanding of the main observations is the following: (1) high sequence and structure diversity of the switch region of all GTPases, (2) high conservation of SRP GTPases and their RNA-binding site, and (3) presence of OB-fold as a second domain of ribosome-associated GTPases. First, I failed to see OB-fold in some proteins, like EF2. In fact SCOP OB-fold domain is absent in all PDB proteins listed in Figure 1, though other beta-barrels with 6 strands and a greek-key topology are present in these structures. Authors may want to provide methods/databases used to derive domain annotation. Second, it was hard for me to establish connections between presented results and some of conclusions. For example, a statement that certain domains of EFG/EF2 and OB-fold domain can potentially mimic the anti codon tRNA stem is unsupported while it can be easily tested by structural superposition.

My recommendation to the authors to publish the manuscript after introducing changes aimed to (i) clarify and summarize Results section and (ii) establish stronger connections to conclusions.

Authors' response: The OB fold is defined in "OB-fold domains: a snapshot of the evolution of sequence, structure and function" by Vickery Arcus (Current Opinion in Structural Biology Volume 12, Issue 6, 2002, pp 794-801) and is found as the second domain of all the ribosomal GTPases in Figure 1. We deleted the speculation as to the general mimicry of the OB-domain.

The OB domain of IF1 does interact with the mRNA codon and that of EFTu with the CCA as referenced. See:Trends in Biochem (2003) v. 28, p 434, Gregers R. Andersen, Poul Nissen and Jens Nyborg.

Chris Sander: This reviewer provided no comments for publication.
